# Metabolic and transcriptome analysis of dark red taproot in radish *(Raphanus sativus L*.*)*

**DOI:** 10.1371/journal.pone.0268295

**Published:** 2022-05-10

**Authors:** Shuangping Heng, Changbin Gao, Mengdi Cui, Jing Fu, Sujing Ren, Kaiyun Xin, Congan He, Aihua Wang, Liping Song, Liguang Tang, Bincai Wang, Xueli Zhang

**Affiliations:** 1 College of Life Science, Xinyang Normal University, Xinyang, P.R. China; 2 Wuhan Vegetable Research Institute, Wuhan Academy of Agriculture Science and Technology, Wuhan, P.R. China; Huazhong University of Science and Technology, CHINA

## Abstract

The red color in radish taproots is an important quality index and is mainly affected by anthocyanins. However, the metabolite components and gene expression underlying dark red taproot color formation in radish remain elusive. In this study, the metabolites and gene expression patterns affecting anthocyanin biosynthesis were monitored in the dark red taproots. Comparative analysis of anthocyanin metabolites between dark red taproots and white taproots indicated that pelargonin and pelargonidin 3-O-beta-D-glucoside were the most promising dark red pigments responsible for the coloration of the taproots. Transcriptomic analysis of gene expression between dark red taproots and white taproots revealed that most of genes involved in the anthocyanin biosynthesis pathway were up-regulated in dark red taproots. In particular, *RsCHS* and *RsDFR* were the two most up-regulated genes in the dark red taproots. Moreover, the higher coexpression of two R2R3-Myb transcription factors, *RsMYB1* and *RsMYB2*, may contribute to dark red color formation. Our work documents metabolomic and transcriptomic changes related to the dark red color formation in taproots radish and provides valuable data for anthocyanin-rich radish breeding.

## Introduction

Flavonoids are commonly known as secondary plant metabolites and are synthesized by both colorless compounds and colored pigments [1]. The colored pigments mainly include anthocyanins, polymeric phlobaphenes and proanthocyanidins. In plants, anthocyanins are responsible for producing different colors, including orange, blue, and red [2]. The colors of various vegetables, fruits, and flowers are the result of specific flavonoid compounds. Anthocyanin, which is one of the most well-known flavonoid members, has been reported in many different plants. The anthocyanin derivatives of delphinidin, petunidin, and malvidin are sources of dark red colors, whereas the derivatives of cyanidin and pelargonidin are the main pigments in bright-red-colored fruits. Many genes and proteins involved in anthocyanin biosynthesis and modification have been studied. The anthocyanin metabolic pathway is regulated by different genes and gene regulatory networks in different plants. Environmental factors also affect the quantitative and qualitative composition of anthocyanins [3].

Metabolomics analysis is used as a powerful and high-throughput approach to identify the main nutrient in many plants. A total of 67 anthocyanins, 102 flavonol glycosides, and 40 hydroxycinnamic acid derivatives were identified in red mustard greens (*Brassica juncea* Coss variety) [4]. Twenty anthocyanin compounds have been identified and quantified in purple and reddish pericarp sweetcorn [5]. Different metabolic profiles between yellow- and black-seeded rapeseed were identified, and the related genes involved in the proanthocyanidin pathway had lower expression in yellow-seeded rapeseed [6]. Transcriptome analysis has also been used widely to identify DEGs involved in anthocyanin biosynthesis [7]. Integrated metabolomics and transcriptomics analysis have been used to identify novel genes involved in flavonoid biosynthesis in *Arabidopsis* [8]. Transcriptomic and metabolomic studies revealed that the main genes involved in flavonoid biosynthesis were up-regulated in red kale compared with green kale [2]. Therefore, the combination of metabolic and transcriptomic methods could better elucidate the metabolomic profiles and gene expression profiles of different biological tissues.

Radish (*Raphanus sativus* L.; 2n = 18) belongs to the *Raphanus* genus in the *Brassicaceae* family. Radish is a very important biennial root vegetable worldwide. Moreover, it is mainly planted in Asian countries, especially in China, Japan, and Korea.

The seedling and taproot of radish are commonly harvested as vegetables, and the radish seeds can also be used for edible oil [9]. It is an important source of nutrients and antioxidants to human diets. Radish color varies from white to red, purple, yellow, green or black skin [10, 11]. Anthocyanins are important water-soluble pigments in radish that are mainly present in the leaves, stems, flowers and taproots. Dark red taproots play an important role in the taste and quality of radish. The anthocyanin concentrates extracted from red radish can often be used as natural food colorants. To date, many studies have focused on the anthocyanin biosynthesis in radish. Pelargonidin is the major anthocyanin from red-skinned or red-fleshed radish [12]. A single dominant gene, *RsMyb1*, positively regulates radish root skin color formation [13, 14]. The R2R3-MYB transcription factor *RsMYB90* controls the red skin color of taproots [15]. The miRNAs and their targeted transcription factors, which are related to anthocyanin biosynthesis, were identified in carmine radish [16]. *RsMATE* genes have been reported to be involved in anthocyanin transport in radish [17]. The up-regulation of MYB1/2/75, bHLH (TT8), WD40 and key enzymes involved in the anthocyanin biosynthesis pathway may contribute to red skin color formation in radish [10]. De novo transcriptome analysis revealed that the *RsDFR1*, *RsDFR2* and *RsFLS* genes might act as key regulators in the anthocyanin biosynthesis pathway in HongXin radish [18]. RNA-seq has been used to discover genes related to anthocyanin accumulation in red and purple color radish [19–21]. RsMYB1a interacts with RsbHLH4 and activates the expression of *RsbHLH4* to regulate anthocyanin accumulation in radish taproots [22]. RsMYB1.3 could interact with RsTT8 [23] to activate the expression of *RsTT8* and *RsUFGT* to produce edible red pigment in Yunnan red radish [24]. However, the transcriptome and metabolome underlying dark red taproot color formation in radish are still unknown. In this study, metabolomics integrated with transcriptomics was employed to clarify the mechanism of anthocyanin biosynthesis and metabolic pathways in dark red radish taproots. This study provides valuable data for further elucidating the relationship between metabolites and gene regulation in dark red radish taproots.

## Material and methods

### Plant materials

Two radish inbred lines, ZIXIN and BAIXIN, with dark red and white taproots, were grown in a greenhouse at the Wuhan Academy of Agriculture Science (Wuhan, China). Flesh samples from the taproots were collected 60 days after sowing. Samples were collected by crosscutting the middle of the radish with three biological repeats. Then, these samples were frozen in liquid nitrogen and stored at -80°C immediately for RNA extraction and metabolic profiling.

### Extraction and determination of anthocyanin content

About 0.1 g taproots were mixed into the liquid with 95% ethanol and 1.5 M HCl (4:1, v/v) in the dark for about 24 h with three biological replicates each. Then, the extracts were centrifuged at 12,000 g for 10 min at 4°C [7]. The anthocyanin content was measured with a Thermo ScientificTM NanoDropTM One with absorption at 530 nm and calculated with previous method [25]. Independent samples T-test is used to compare the means of the two taproot samples with significant differences at P < 0.01.

### Extraction of metabolites from dark red and white taproots

The freeze-dried root was crushed using a mixer mill (MM 400, Retsch) with a zirconia bead for 1.5 min at 30Hz. One hundred milligrams of powder from each sample was extracted overnight with 1.0 ml 70% aqueous methanol at 4°C. Following centrifugation at 10,000 g for 10 min, the extracts were absorbed (CNWBOND Carbon-GCB SPE Cartridge, 250 mg, 3 ml; ANPEL, Shanghai, China, www.anpel.com.cn/cnw) and filtered with a nylon syringe filter (SCAA-104, 0.22 μm pore size; ANPEL, Shanghai, China, http://www.anpel.com.cn/) before LC-MS analysis. Metabolite identification and quantification were performed at MetWare Biotechnology Co., Ltd. (Wuhan, China). Three biological replicates each were used for the metabolic studies of two radish varieties. The anthocyanin contents of the taproot flesh of the ZIXIN and BAIXIN plants were measured as previously described [25].

### HPLC conditions

The sample extracts were analyzed by using an LC-ESI-MS/MS system (HPLC, Shim-pack UFLC SHI MADZU CBM30A system, www.shimadzu.com.cn/; MS, Applied Biosystems 6500 Q TRAP, www.appliedbiosystems.com.cn/). The analytical conditions were as follows: HPLC column, Waters ACQUITY UPLC HSS T3 C18 (1.8 µm, 2.1 mm*100 mm); solvent system, water (0.04% acetic acid): acetonitrile (0.04% acetic acid); gradient program, 95:5 V/V at 0 min, 5:95 V/V at 11.0 min, 5:95 V/V at 12.0min, 95:5 V/V at 12.1min, and 95:5 V/V at 15.0 min; flow rate, 0.40 ml/min; temperature, 40°C; and injection volume: 2 µl. The effluent was alternatively connected to an ESI-triple quadrupole-linear ion trap (Q-TRAP)-MS.

### ESI-Q TRAP-MS/MS

Linear ion trap (LIT) and triple quadrupole (QQQ) scans were acquired on a triple quadrupole-linear ion trap mass spectrometer (Q-TRAP), API 6500 Q TRAP LC/MS/MS System, equipped with an ESI Turbo Ion-Spray interface, operating in positive ion mode and controlled by Analyst 1.6.3 software (AB Sciex). The ESI source operation parameters were as follows: ion source, turbo spray; source temperature 500°C; ion spray voltage (IS), 5500 V; ion source gas I (GSI), gas II (GSII), and curtain gas (CUR) were set at 55, 60, and 25.0 psi, respectively. Instrument tuning and mass calibration were performed with 10 and 100 μmol/L polypropylene glycol solutions in QQQ and LIT modes, respectively. QQQ scans were acquired as multiple reaction monitoring (MRM) experiments with collision gas (nitrogen) set to 5 psi. The declustering potential (DP) and collision energy (CE) for individual MRM transitions were determined with further DP and CE optimization. A specific set of MRM transitions was monitored for each period according to the metabolites eluted within this period.

### RNA-seq and gene annotation

Total RNA from the dark red and white taproots was extracted by the TRIzol (Invitrogen, USA) method. RNA isolation and purification, and cDNA library construction and sequencing were performed as described previously [7]. RNA quantity and quality were determined by a NanoDrop ND1000 spectrophotometer (NanoDrop Technologies, Wilmington, DE, USA) and an Agilent Bioanalyzer 2100 system (Agilent Technologies, Palo Alto, CA, USA). RNA integrity was determined by 1% agarose gel electrophoresis, and the RNA concentration was adjusted for uniformity. mRNA was isolated from total RNA using Oligo (dT) magnetic beads. Then, mRNA was fragmented randomly by ultrasonication. Using the fragmented mRNA as a template, a 6-base random primer was used to synthesize the first strand of cDNA in the M-MuLV reverse transcriptase system. Subsequently, the RNA strand was degraded by RNaseH, and the second strand cDNA was synthesized from dNTPs under the DNA polymerase I system. The purified double-stranded cDNA was end-repaired, a tail was added, and the sequencing linker was ligated. The cDNA of approximately 200 bp was selected with AMPure XP beads, and the PCR amplification product was purified again by using AMPure XP beads to obtain a library. The library preparations were sequenced on an Illumina HiSeq platform. The RNA seq are available used for the analysis of DEGs in this study were deposited in NCBI with accession number: PRJNA810281 and PRJNA810914. The sequence data were mapped to the *Raphanus sativus* genome (http://radish.kazusa.or.jp/).

### Analysis of differentially expressed genes (DEGs)

FPKM (Fragments Per Kilobase of transcript per Million mapped reads) was used to estimate the expression of different genes. The FPKM was used to calculate the correlation coefficients of the samples in and between different groups. DESeq2 was used to identify DEGs between ZIXIN and BAIXIN by calculating the reads from each gene with three biological replicates. The Benjamini-Hochberg method was used to perform multiple hypothesis test correction on the hypothesis test probability (P-value) to obtain the false discovery rate (FDR).

### Real-time PCR

First-strand cDNA was synthesized by using the PrimeScript™ RT Reagent Kit (RR037A) with gDNA Eraser (TaKaRa, Japan). The relative gene expression assay was carried out by using the CFX96 Real-Time System (Bio-Rad, USA). The transcript levels of DEGs were analyzed using the 2^-ΔΔCT^ method [26]. The *RsActin* gene was used as an internal control. The reactions were performed with three biological replicates. The primers used for the real-time PCR and semi-quantitative RT-PCR assays are listed in S1 Table. Significant differences were determined with SPSS software by using two-tailed unpaired Student’s t-tests. *P* values<0.05 were considered to represent statistically significant differences.

## Results

### Phenotype differences between the dark red and white radish taproots

**Fig 1:**
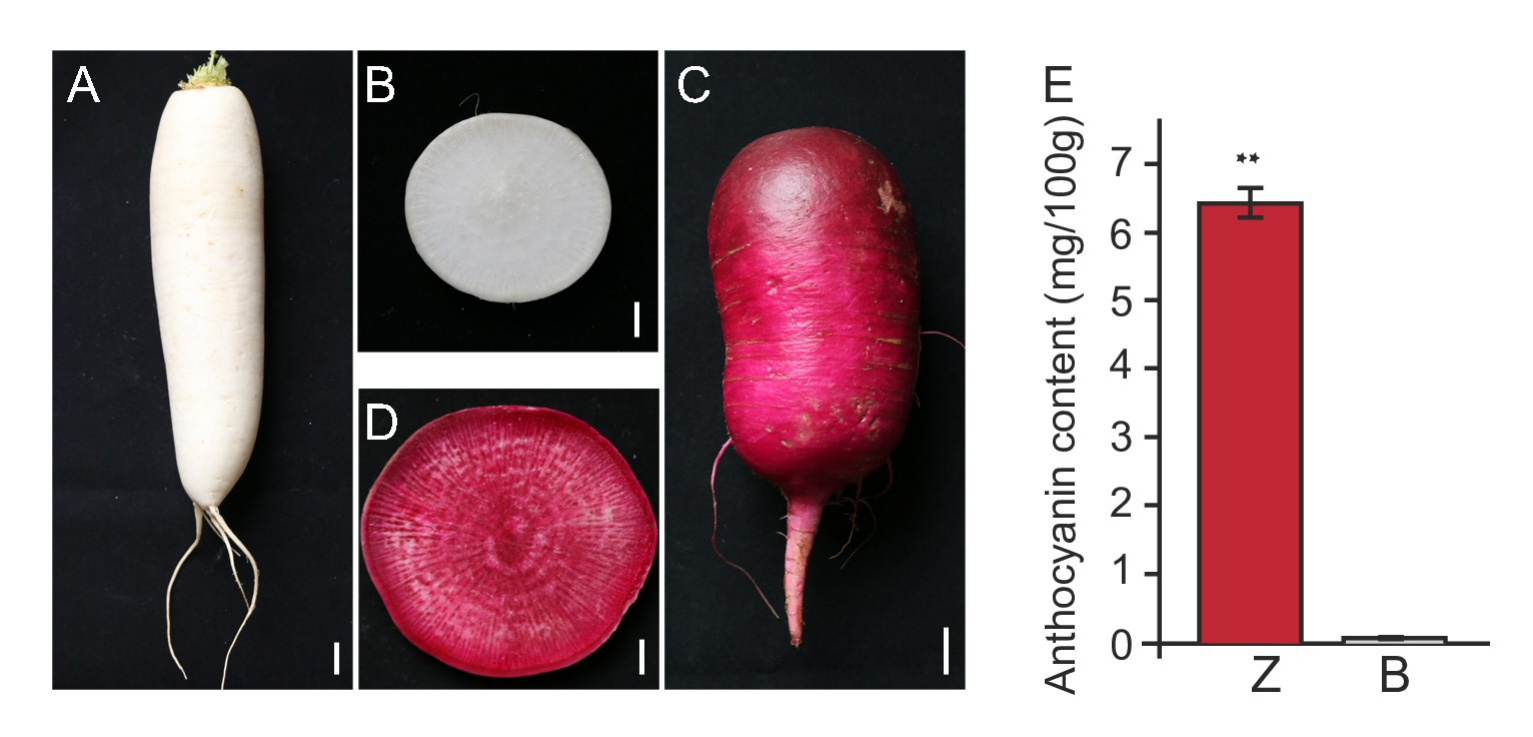
The phenotype of dark red and white radish taproots. **A.** The epidermis of a white radish taproot; **B.** Transection of a white radish taproot; **C.** The epidermis of a dark red radish taproot; **D.** Transection of a dark red radish taproot; **E.** Anthocyanin levels in dark red and white radish taproots. A, C Scale bars 2 cm; B, D Scale bars 1 cm. B:BAIXIN; Z:ZIXIN. Error bars indicate standard deviations for three biological replicates. ** reveals significantly different with P < 0.01.

With the radish taproots development, no dark red or red color appeared in the skin and flesh of BAIXIN taproot (Fig 1A and 1B). The skin and flesh of ZIXIN taproots were dark red when compared with those of BAIXIN taproots (Fig 1C and 1D). The red color is the result of high levels of anthocyanin accumulation in radish. Dark red taproots showed significantly higher anthocyanin content than that of the white taproots (Fig 1E). The higher anthocyanin content contributes to dark red color formation in taproots.

### Metabolic differences among the radish

**Fig 2:**
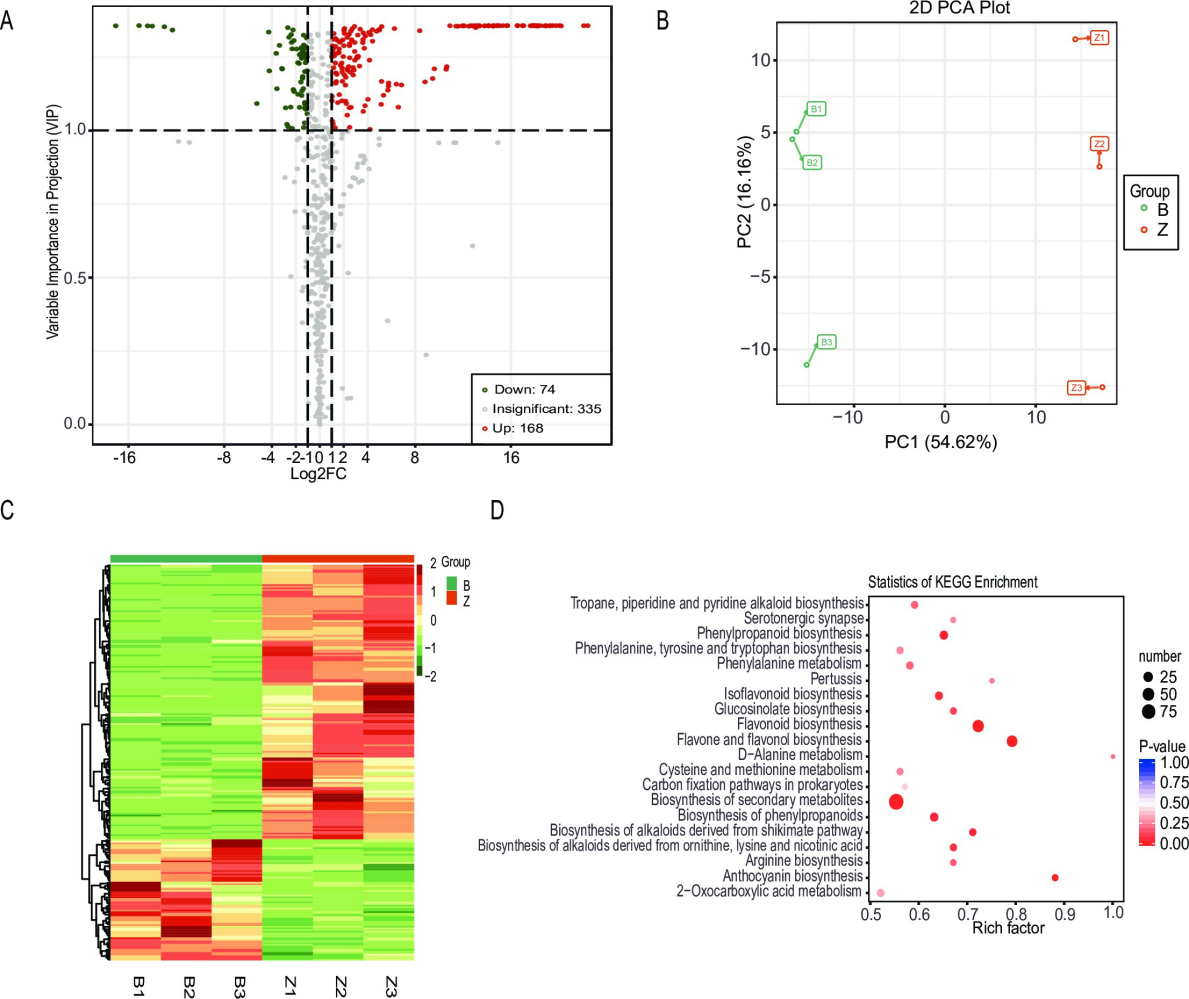
Metabolic differences between dark red and white radish taproots. **A.** Volcano plots of different metabolites between the dark red and white taproots; **B.** Principal component analysis (PCA) score map with data from the dark red and white samples; **C.** A hierarchical clustering graph based on the expression values of all significantly different metabolites identified in dark red and white taproots; **D.** Kyoto Encyclopedia of Genes and Genomes (KEGG) enrichment of these different metabolites.

To profile the metabolic changes between the dark red and white radish taproots, metabolome analysis was performed by using UPLC-MS/MS. A total of 577 different metabolites were detected (Fig 2A). After a comparative analysis of these different metabolites between the ZIXIN and BAIXIN, a total of 242 differentially accumulated metabolites (DAMs) were found (S2 Table). Among them, 168 metabolites showed a significantly higher content in ZIXIN than in BAIXIN, and 74 metabolites showed a significantly lower content in ZIXIN than in BAIXIN. To compare the metabolite composition in ZIXIN and BAIXIN, a principal component analysis (PCA) plot of the metabolites was conducted. The metabolites of these two radishes were separated based on the PC1 and PC2 score plots (Fig 2B). The principal components PC1 (54.62% variance) and PC2 (16.16% variance) were separated into B group (BAIXIN) and Z group (ZIXIN). Cluster analysis showed that the DAMs were clustered into distinct groups (Fig 2C).

To further explore the biological pathways, the DAMs were annotated using the Kyoto Encyclopedia of Genes and Genomes (KEGG). Among them, D-Alanine metabolism, anthocyanin biosynthesis, and flavone and flavonol biosynthesis were the most common significant typical pathways changed between ZIXIN and BAIXIN (Fig 2D). There were 10 different anthocyanin metabolites detected in dark red and white radish taproots (Table 1), 8 of them accumulated more in ZIXIN. The three most abundant anthocyanin metabolites in dark red-fleshed radish were pelargonidin, pelargonidin 3-O-beta-D-glucoside and pelargonidin 3-O-malonylhexoside.

**Table 1:** Different anthocyanin metabolites were detected in dark red and white radish taproots.

Compounds	B1	B2	B3	Z1	Z2	Z3	Fold Change (FC)	Log2FC	Type
Pelargonin	9.00E+00	9.00E+00	9.00E+00	4.34E+07	5.39E+07	5.63E+07	5.69E+06	2.24E+01	up
Pelargonidin 3-O-beta-D-glucoside	9.00E+00	9.00E+00	9.00E+00	3.55E+07	3.12E+07	5.52E+07	4.51E+06	2.21E+01	up
Pelargonidin 3-O-malonylhexoside	9.00E+00	9.00E+00	9.00E+00	5.92E+06	1.33E+07	1.34E+07	1.21E+06	2.02E+01	up
Cyanidin O-acetylhexoside	9.00E+00	9.00E+00	9.00E+00	3.38E+06	2.62E+06	2.43E+06	3.12E+05	1.83E+01	up
Cyanidin	9.00E+00	9.00E+00	9.00E+00	1.88E+06	2.18E+06	3.77E+06	2.90E+05	1.81E+01	up
Cyanidin 3-O-glucoside (Kuromanin)	9.00E+00	9.00E+00	9.00E+00	2.52E+06	3.07E+06	1.74E+06	2.71E+05	1.81E+01	up
Cyanidin 3,5-O-diglucoside (Cyanin)	9.00E+00	9.00E+00	9.00E+00	1.85E+06	2.79E+06	2.33E+06	2.58E+05	1.80E+01	up
Malvidin 3,5-diglucoside (Malvin)	9.00E+00	9.00E+00	9.00E+00	1.03E+05	8.11E+04	7.00E+04	9.41E+03	1.32E+01	up
Malvidin	1.21E+05	1.17E+05	1.83E+05	8.35E+04	5.15E+04	5.51E+04	4.52E-01	-1.15E+00	down
Malvidin 3-acetyl-5-diglucoside	6.00E+04	1.25E+05	3.64E+04	9.00E+00	9.00E+00	9.00E+00	1.22E-04	-1.30E+01	down

Note: Relative quantification was calculated by the area of each individual peak. FC: Fold Change

### RNA-seq and identification of DEGs between BAIXIN and ZIXIN

**Fig 3:**
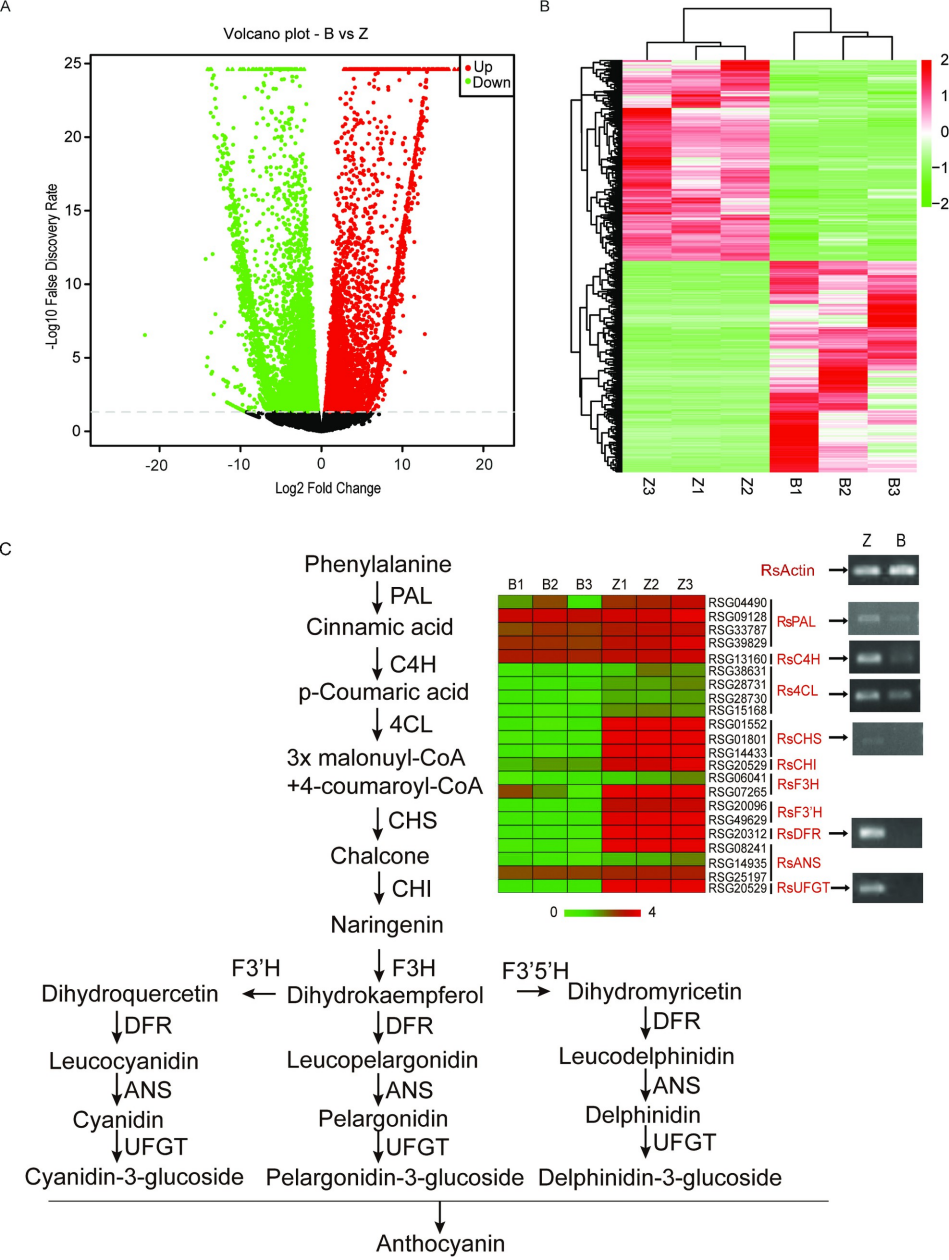
Expression profiles of differentially expressed genes (DEGs) in dark red and white radish taproots. **A.** Volcano plot showing significant DEGs between the dark red and white taproots; **B.** Hierarchical clustering graph based on the DEGs between dark red and white taproots. **C.** DEGs involved in the anthocyanin biosynthesis pathway selected from the transcriptome data and RT-PCR analysis of the key enzymes involved in anthocyanin biosynthesis. Abbreviations: ANR, anthocyanidin reductase; ANS, anthocyanidin synthase; AS, aurone synthase; C4H, cinnamate 4-hydroxylase; CHI, chalcone isomerase; 4CL, 4-coumarate coenzyme A ligase; CHR, chalcone reductase; CHS, chalcone synthase; DFR, dihydroflavonol reductase; F3H, flavanone 3-hydroxylase; F3’H, flavonoid 3’-hydroxylase; F3’5’H, flavonoid 3’-5’-hydroxylase.

To better understand the gene expression profile of anthocyanin biosynthesis in dark red radish taproots, *RNA-seq* was employed to compare the DEGs between BAIXIN and ZIXIN. A total of 185,951,818 and 176,890,366 raw reads were obtained from the BAIXIN (B) and ZIXIN (Z) libraries, respectively. More than 92.64% of the reads showed a map quality score >30 (Q30). After filtered the low-quality data, a total of 358,081,402 clean reads were obtained. These sequences were aligned to the *Raphanus sativus* genome (http://radish.kazusa.or.jp/). The rate of uniquely mapped reads for each library ranged from 67.87% to 81.41% (S3 Table). DEGs were estimated by calculating the FPKM values (log2-fold change ≥1 and FDR≤0.01). A total of 9,266 DEGs were identified between dark red taproots and white taproots (S4 Table). There were 4,517 up-regulated and 4,749 down-regulated genes (Fig 3A). These DEGs were clustered into distinct groups (Fig 3B). It is well known that the MYB-bHLH-WD40 (MBW) transcription factor complex could increase anthocyanin synthesis by regulating genes involved in the anthocyanin synthesis pathway. Therefore, we mainly focused on genes involved in the anthocyanin synthesis pathway and the MYB-bHLH-WD40 (MBW) transcription factors, which regulate anthocyanin synthesis among these DEGs.

### Comparative analysis of key enzymes involved in anthocyanin biosynthesis

It has been widely known that genes involved in anthocyanin biosynthesis pathway affect color change in different plants. Therefore the genes, which encode key enzymes involved in anthocyanin biosynthesis, were selected from the DEGs between dark red and white taproots (S5 Table). Among them, there is only one copy of *RsC4H* (cinnamate 4-hydroxylase), *RsCHI* (chalcone isomerase), *RsDFR* (dihydroflavonol 4-reductase) and *RsUFGT* (UDP-glucose flavonoid 3-O-glucosyl transferase) in radish. The genes *RsF3H* (flavanone 3-hydroxylase) and *RsF3’H* (flavanone 3’-hydroxylase) have two copies each. *RsCHS* (chalcone synthase) and *RsANS* (anthocyanidin synthase) genes with 3 copies each. *RsPAL* (phenylalanine ammonia-lyase) and *Rs4CL* (4-coumaryol CoA ligase (4CL) have 4 copies each. As expected, these genes were up-regulated in dark red taproots (Fig 3C). *RsCHS* (RSG01552), *RsDFR* (RSG20312), *RsANS* (RSG08241), and *RsUFGT* (RSG45505) were the most up-regulated genes in dark red taproots. The higher expression of these genes may contribute to the dark red coloration formation in ZIXIN taproots.

**Fig 4:**
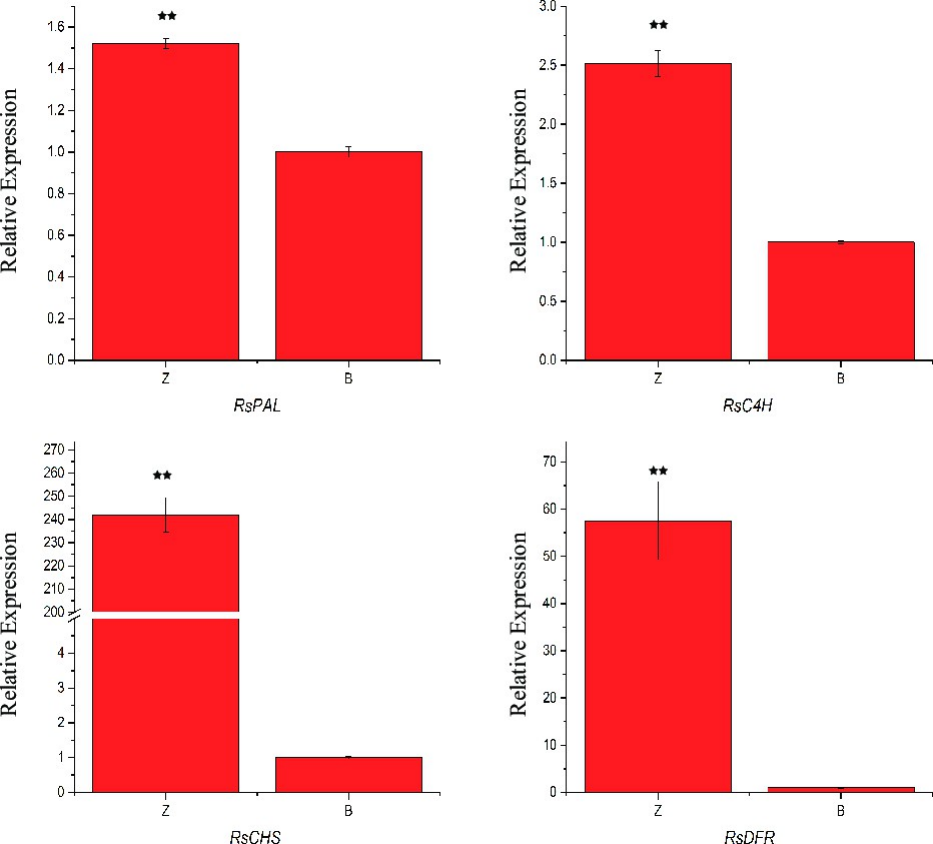
qRT-PCR analysis of the transcript expression levels of the *RsPAL*, *RsC4H*, *RsCHS* and *RsDFR* genes between dark red and white radish taproots. Error bars indicate standard deviations for three biological replicates. ** reveals significantly different with P < 0.01.

To verify the reliability of the gene expression results revealed by RNA-seq between dark red and white taproots, some of the key enzymes involved in anthocyanin biosynthesis in dark red radish taproots were selected for RT-PCR (Fig 3C). Then, four DEGs (*RsPAL*, *RsC4H*, *RsCHS* and *RsDFR*) responsible for anthocyanin biosynthesis were selected for qPCR (Fig 4). The transcript expression pattern of all these DEGs was consistent with our transcriptome data. Our study further confirmed that up-regulation of key enzymes involved in anthocyanin biosynthesis induced the dark red color formation.

**Transcript expression** of RsMYB1 and RsMYB2 in dark red radish taproots.

To further explore key transcription factors involved in the dark red color formation, among these DEGs, a total of 212 up-regulated transcription factors in dark red radish taproots were identified. These TFs were divided into more than 37 different types. Among them, the MYB- and WRKY-type TFs were the most abundant TFs (S6 Table).

**Fig 5:**
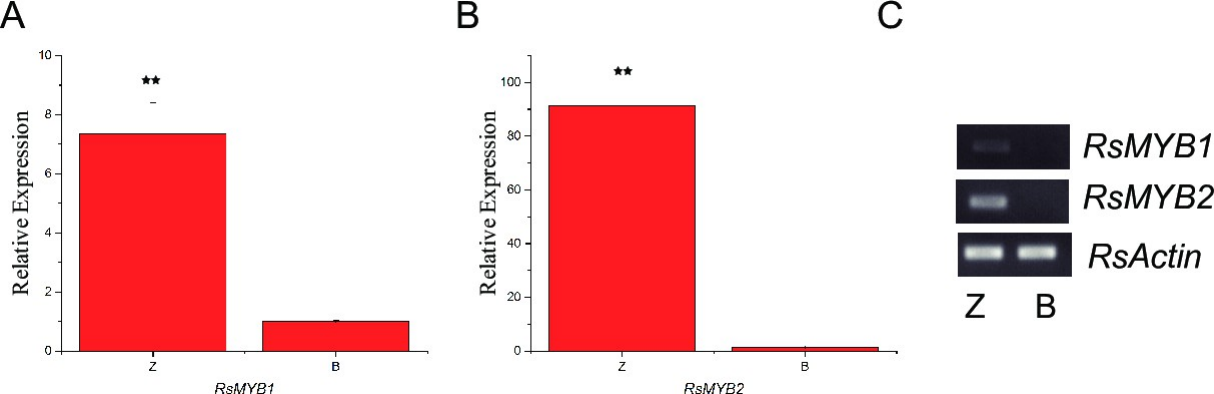
qRT-PCR and RT-PCR analysis of *RsMYB1* and *RsMYB2* between dark red and white radish taproots. Error bars indicate standard deviations for three biological replicates. ** reveals significantly different with P < 0.01.

Up-regulation some of the key transcription factors such as MYB and bHLH could promote leaf color change in plants. A total of 25 MYB and 9 bHLH TFs identified from the 212 up-regulated transcription factors showed higher expression in the dark red taproot. Among them, *RsMYB1* (RSG33469) and *RsMYB2* (RSG19108) were more highly expressed in dark red taproots than in white taproots. And RSG22617, which encode a bHLH TF, showed significantly higher expressed in dark red taproots. qPCR and RT-PCR experiments further confirmed that *RsMYB1* and *RsMYB2* were more highly expressed in dark red taproots (Fig 5). The transcript expression of *RsMYB2* was also significantly higher than that of *RsMYB1*. Our results highlighted that both *RsMYB1* and *RsMYB2* activated the anthocyanin biosynthesis in the dark red taproots.

## Discussion

It is widely known that anthocyanin and anthocyanin derivatives are the main sources of dark purple colors formation in plants. Although 16 different anthocyanins were identified in the radish cultivar Man Tang Hong, which has red flesh and green skin, 15 of them were pelargonin derivatives [27]. In our study, the dark red radish inbred line ZIXIN showed red flesh and red skin. Anthocyanin biosynthesis, flavone and flavonol biosynthesis related metabolites were identified by metabolomics analysis. Anthocyanins including 4 types of cyanidin and their derivatives and 3 types of malvidin and their derivatives, were identified in dark red radish. These anthocyanin metabolites contribute to the taproot dark red color formation.

Recent studies have shown that glutathione S-transferases (GSTs) are responsible for the transport of anthocyanins from the cytosol into the vacuole. It has been suggested that *AtTT19* functions as a carrier of anthocyanins in *Arabidopsis* [28]. Transcriptome analysis showed that the late anthocyanin biosynthetic genes (LBGs), including *DFR* and *ANS*, were highly up-regulated in purple leaves of *Brassica* [29]. In apple, MdGSTF6 can be activated by MdMYB1 to transport and accumulate anthocyanin [30]. In strawberry, RAP, which encodes a GST transporter of anthocyanins, could be modified to change the fruit color [31]. The *CsGSTF1* gene, which is a functional orthologous gene of *AtTT19*, is associated with anthocyanin accumulation in purple tea [32]. In this study, transcriptome analysis showed that *RsTT19* was also up-regulated in the dark red radish taproots. Up-regulation of *RsTT19* may affect the dark red color formation in radish.

The anthocyanin biosynthesis pathway has been well studied in plants [33, 34]. The pathway is regulated by the MYB-bHLH-WD40 (MBW) ternary complex [35]. The anthocyanin-promoting R2R3-MYB gene *RsMyb1* (KR706195) was first cloned by RACE from the radish cultivar “Bordeaux” [14]. *RsMyb1* (Rs388430), which encodes a MYB114-like transcription factor, was further confirmed to determine the red radish external skin color of radish roots [13]. The *RsMYB90* gene was also reported to regulate anthocyanin accumulation in red-skinned radish [15]. Moreover, the CACTA transposon in the promoter of *RsMYB1* is required for the red-fleshed phenotype of radish. DNA methylation of the *RsMYB1* (RSG33469) promoter inhibited anthocyanin biosynthesis in white-fleshed mutants. Only *RsMYB1*, but not *RsMYB2* was expressed in the Xinlimei landrace, which has red flesh and green skin [36]. However, in our study, TFs such as *RsMYB1* and *RsMYB2* contributed to the accumulation of anthocyanin metabolites (pelargonidin, pelargonidin 3-O-beta-D-glucoside and pelargonidin 3-O-malonylhexoside) and contributed to the dark red color of radish taproots.

Together, the metabolomic analysis identified the main anthocyanin in dark red radish taproots. The transcriptomic analysis identified the key genes involved in anthocyanin biosynthesis. By combining the metabolomics and transcriptomics data, the metabolites and genes involved in dark red color formation in taproots were identified. Our results may provide new insights into dark red taproot color formation at the metabolic and molecular levels.

## Conclusions

By combining metabolomics with transcriptomics data, the different metabolites and DEGs related to the anthocyanin biosynthetic pathway in dark red taproots were identified. Most of the key enzymes involved in anthocyanin biosynthesis were up-regulated in dark red taproots. Pelargonin-based and cyanidin-based anthocyanin metabolites were detected in dark red radish taproots. The anthocyanin metabolite change was consistent with the anthocyanin biosynthetic gene expression. Gene expression related to the anthocyanin biosynthesis pathway was highly correlated with specific anthocyanin metabolites in dark red taproots. Our study may guide the improvement of the commercial and nutritional quality of radish.

## Supporting information

S1 Table Primers used for real-time PCR and semi-quantitative RT-PCR. (XLSX)

S2 Table Differentially accumulated metabolites detected between dark red and white radish taproots. (XLSX)

S3 Table Summary of RNA-seq data from dark red and white taproots in radish. (XLSX)

S4 Table RNA-seq DEGs identified between dark red taproots and white taproots. (XLSX)

S5 Table Key enzymes involved in anthocyanin biosynthesis. (XLSX)

S6 Table Up-regulated transcription factors in dark red radish taproots. (XLSX)

S1 Raw images (PDF)
