# Supplementary figures and images for: Metabolic and transcriptome analysis of dark red taproot in radish (Raphanus sativus L.)
Source: PLoS One. 2022 May 10;17(5):e0268295. doi: 10.1371/journal.pone.0268295 (PMC9089891; doi:10.1371/journal.pone.0268295)

Fig 3. C Raw images

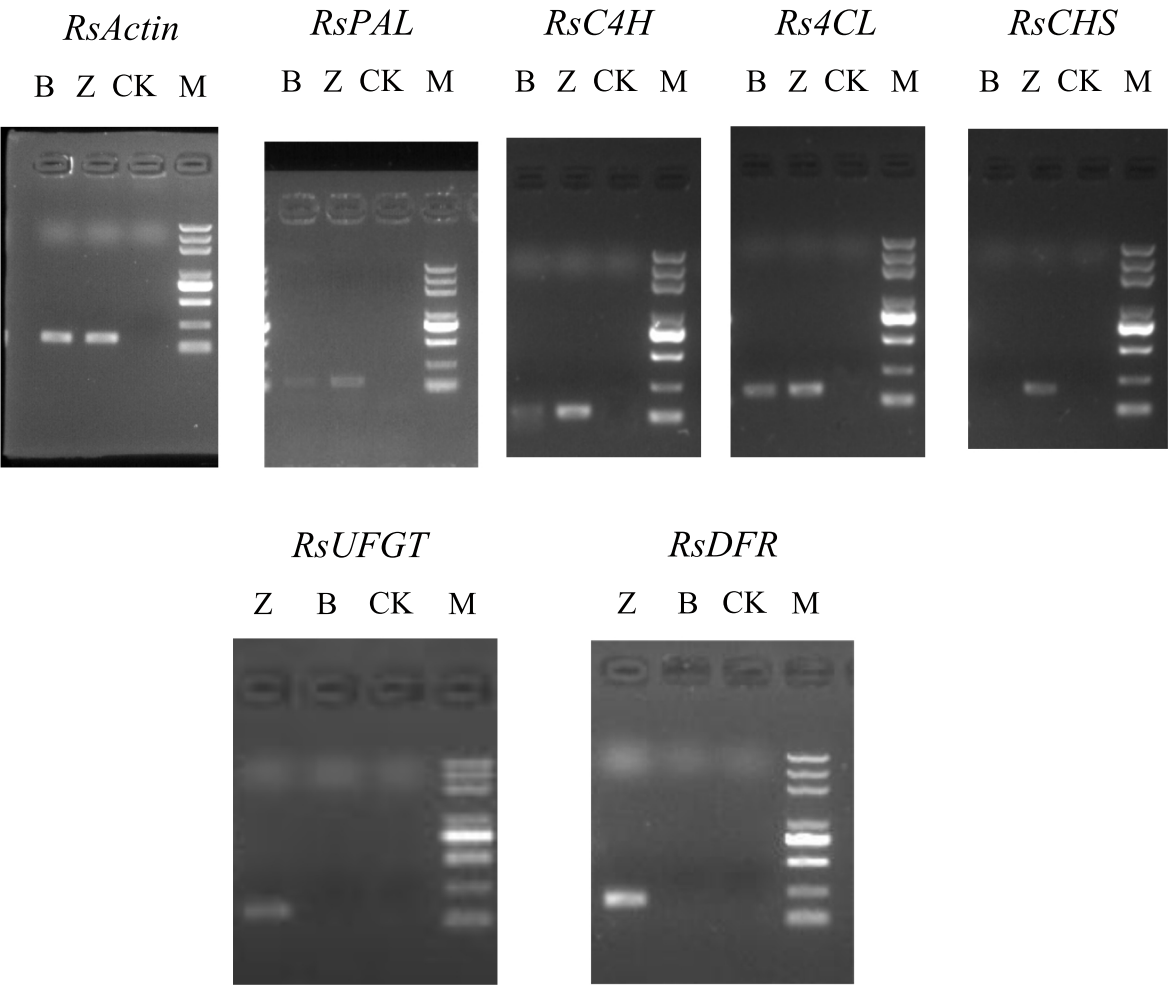

Fig 5. C Raw images

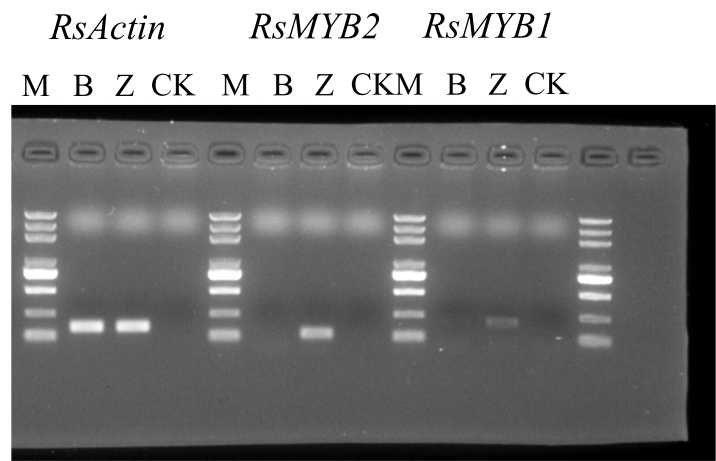

Supplement: S1 Raw images — (PDF) [file pone.0268295.s007.pdf]
